# Clinical and Experimental Immunomodulation 2014

**DOI:** 10.1155/2015/758328

**Published:** 2015-04-28

**Authors:** Lenin Pavón, Oscar Bottasso, Hugo Besedosky, Roger Loria, Marco A. Velasco-Velázquez

**Affiliations:** ^1^Department of Psychoimmunology, National Institute of Psychiatry “Ramón de la Fuente”, Calzada México-Xochimilco 101, Colonia San Lorenzo Huipulco, Tlalpan, 14370 Mexico City, DF, Mexico; ^2^Instituto de Inmunología, Facultad de Ciencias Médicas, Universidad Nacional de Rosario, Santa Fe 3100 CP, 2000 Rosario, Argentina; ^3^Research Group Immunophysiology, Institute of Physiology and Pathophysiology, Philipps University, 35037 Marburg, Germany; ^4^Department of Microbiology, Immunology, Virginia Commonwealth University, 1101 E. Marshal Street, Richmond, VA 232980678, USA; ^5^School of Medicine, National Autonomous University of Mexico, Avenida Universidad 3000, Coyoacan, 04510 Mexico City, DF, Mexico

The proper function of cells, receptors, and soluble mediators involved in the immune response is associated with an individual's health. However, hyper- or hyporeactive immune responses are associated with a broad spectrum of diseases, such as chronic inflammatory diseases, infections, allergies, autoimmune diseases, and immunodeficiencies. Several factors could contribute to the deregulation of the immune response, some of them involving genetic and environmental factors as well as an individual's perception. In recent years, immunomodulatory effects of apparently unrelated molecules such as hormones and neurotransmitters have been reported and their potential therapeutic applications have been identified. Thus, the investigation of molecules and compounds with immunomodulatory properties holds the promise of the development of new therapeutic approaches for a wide range of ailments. This journal has inaugurated an annual issue to emphasize the leverage that knowledge on clinical and experimental immunomodulation has come to attain. This special issue is the second volume of such series.

In this issue, the reader will find thirteen experimental approaches analyzing several aspects of immunomodulation. Four of them focus on the immunomodulatory effects of microorganisms. For example, the work of L. Chen et al. shows modulatory effects of* Lactobacillus acidophilus* on the IL-23/Th17 immune axis in a murine experimental colitis model. Their results show that the administration of* L. acidophilus* suppressed Th17 cell-mediated secretion of the proinflammatory cytokine IL-17 through downregulation of IL-23 and TGF-*β*1 expression and downstream phosphorylation of STAT3. In this same sense, C. C. Squaiella-Baptistão et al. analyzed the mechanisms accounting for the Th2 response elicited by inactivated* P. acnes* or its soluble polysaccharide in an ovalbumin immediate hypersensitivity model, showing that the potentiating or suppressing effects on Th2 response seem to be related to the expression of costimulatory molecules or toll-like receptors (TLRs), on antigen presenting cells. The study by T. F. de C. Fraga-Silva et al. provides evidence of the aggravating role of a prior* Candida albicans* infection on the course of an experimentally induced autoimmune encephalomyelitis in female C57BL/6 mice. Manipulated animals display not only a higher clinical score but also a more noticeable expansion of peripheral CD4+ T lymphocytes together with an increased production of TNF-*α*, IFN-*γ*, IL-6, and IL-17 either in the spleen or in CNS. The work by B. Franz et al. compares the features of the experimental infection by* Francisella tularensis* (*Ft*) in wild-type and Fc gamma receptor IIB (Fc*γ*RIIB) knockout mice vaccinated with inactivated* Ft* (i*Ft*), to study the role of Fc*γ*RIIB in downregulation of the immune response after the interaction of Fc*γ*RIIB with immune complexes. While showing no survival differences, i*Ft*-immunized Fc*γ*RIIB KO mice display a better response compared to their wild-type counterparts in terms of specific IgA levels and synthesis of cytokines involved in the inflammatory reaction.

A different set of studies assessed drugs with immunomodulatory effects. The work of M. P. Miranda-Hernández et al. presents a physicochemical and biological characterization, followed by pharmacodynamic and immunogenicity studies in two rituximab containing products, which behaved in a similar manner both physicochemically and functionally. In another work, N. Salinas-Jazmín et al. evaluated the activity of multiple batches of a human dialyzable leukocyte extract (hDLE), a mixture of low-molecular weight peptides released from lysed peripheral blood leukocytes, in a standardized and validated mouse model of cutaneous HSV-1 infection. The activity of all batches of hDLE improved survival from HSV-1 infection, in presence of increased levels of IFN-*γ* and reduced amounts of IL-6 and TNF-*α* in mice sera. Q. Qiu et al. analyze the effectiveness of a short bromocriptine treatment for the prevention of postpartum relapse in systemic lupus erythematosus (SLE) patients. Bromocriptine effectively prevents postpartum hyperprolactinemia and hyperestrogenemia in the first two months after delivery and lowers the relapse rate, showing that bromocriptine may be beneficial for a subset of SLE patients. P. Schafer et al. assess the role of systemic inflammatory biomarkers in the previously reported effect of apremilast in psoriatic arthritis. They found that, in a first phase of treatment, apremilast reduced plasma levels of several Th1 proinflammatory cytokines, especially TNF-*α*. In contrast, longer treatment times reduced the concentration of Th17 cytokines and increased anti-inflammatory mediators. The results show that apremilast induces a new balance in Th1 and Th17 cytokines that partially explain its therapeutic effects.

Two more papers analyze the role of physiological mediators in immune response. S. Muñoz-Cruz et al. demonstrate that histamine release by mast cells following stimulation with IgE or substance P is influenced by sex hormones in a dose- and gender-dependent manner, with female rats being more susceptible to the hormonal influence. E. Carbajal-Franco et al. explore the role of inhibins and activins in the thymic stromal cell differentiation and function. Their findings demonstrate that inhibins modify the relationship between cortical and medullary thymic epithelial cells, further influencing thymocyte selection and generation of natural Treg cells.

This issue also includes experimental approaches for analyzing immune changes during disease. R. Masetti et al. present a retrospective study of a cytokine SNPs panel in transplanted children with acute graft-versus-host disease (aGvHD). Results reveal that SNPs on* IL-10*,* FAS, *and* TLR4* in donors and* IL-18* in recipients are associated with an increased risk of developing aGvHD. F. Morandi et al. evaluated the contribution of tumor cells to the immunosuppression observed in patients with metastatic neuroblastoma. Although they found increased IL-10, ARG1, and CD163 plasma concentration and increased Treg cells in NB patients, the changes were independent of the clinical outcome and therefore cannot be used as prognostic factors. In addition, A. Diaz et al. studied Treg frequency as well as plasma concentrations of cytokines and steroid hormones in tuberculosis (TB) patients. They show that the increased Treg frequency in TB patients compared to healthy controls is further increased by TB-specific therapy. After four months of treatment, patients also display reduced levels of the proinflammatory cytokine IFN-*γ*. These results partially support the hypothesis that Treg cells play a role in counterbalancing an excessive inflammatory response in TB patients.

There are also in this issue eight interesting reviews about agents with immunomodulatory properties. R. Arreola et al. present a wide review about the mechanisms involved in 5-HT-induced immunomodulation and their effects in specific pathologies such as major depression, fibromyalgia, Alzheimer's disease, psoriasis, arthritis, allergies, and asthma. K. J. G. Díaz-Resendiz et al. comment on several lines of evidence pointing out to an affectation of the immune response following exposure to organophosphorus pesticides. The evidence presented suggests that, among the potential mechanisms involved, these pesticides affect neuroimmune communication, particularly the cholinergic branch and the immune system. P. S. de Araújo-Souza et al. reviewed the chromatin status of the* IFN-γ* gene promoter in T CD8^+^ cells and the potential influences of epigenetic modifications and conserved noncoding sequences in the regulation of IFN-*γ* synthesis by these cells. C. Pacheco-Tena and S. A. González-Chávez provide an appealing view on the pathogenesis of several rheumatic diseases. In essence, they point out tissue dysfunction as a critical prerequisite for chronic autoimmunity. Z. Mohammed-Ali et al. present the accumulated evidence about the cross talk between the unfolded protein response (UPR) and NF-*κ*B pathways in chronic kidney disease. They also discuss alternative tools to study this phenomenon as well as the potential therapeutic candidates that are emerging to regulate the UPR. R. Arreola et al. review the stimulating effects of garlic on several immunocompetent cells including macrophages, lymphocytes, NK cells, DCs, and eosinophils. Moreover, this work examines potential avenues for garlic-based modulation strategies in several diseases with an immunological component in its pathogenesis. M. E. Castro-Manrreza and J. J. Montesinos review the effects elicited by mesenchymal stem cells (MSCs) on immune cells, with particular focus on the mechanisms involved, and discuss the potential clinical application of MSCs in immune disorders. Finally, M. Vestita et al. present an extensive review of the literature of the role of vitamin D in atopic dermatitis (AD). The evidence suggests that vitamin D supplementation may elicit a therapeutic effect in AD; however, at present, its administration is only justified in very rare therapy resistant cases.

We hope that our readers will find this special issue enticing and enjoy reading the contributions by all authors, as we have done.

*Lenin Pavón* *Lenin Pavón* *Oscar Bottasso* *Oscar Bottasso* *Hugo Besedosky* *Hugo Besedosky* *Roger Loria* *Roger Loria* *Marco A. Velasco-Velázquez* *Marco A. Velasco-Velázquez*

